# A Case of Daptomycin-Induced Rhabdomyolysis: A Life and Limb Threatening Complication

**DOI:** 10.7759/cureus.38285

**Published:** 2023-04-29

**Authors:** Jenna Gunn, Kelsee K Zajac, Katherine Esser, David Yatsonsky, Paige Chapman

**Affiliations:** 1 Orthopaedic Surgery, University of Toledo College of Medicine and Life Sciences, Toledo, USA

**Keywords:** daptomycin related adverse effect, emergent fasciotomy, adverse drug effect, rhabdomyolysis, acute compartment syndrome

## Abstract

Surgical site infections (SSIs) contribute to patient morbidity and health expenditure. An increasing elderly population, the expanding use of implants in surgical procedures, drug-resistant microorganisms, and patient-related comorbidities all contribute to SSIs. Daptomycin is an antibiotic known to cause rhabdomyolysis, a life-threatening complication that may lead to acute compartment syndrome (ACS). We present a case of a patient treated with daptomycin for a penile-implant infection complicated by rhabdomyolysis and ACS of his bilateral forearms. He underwent emergent fasciotomies and retained function in his upper extremities long-term. It is vital that physicians closely monitor patients treated with IV-daptomycin therapy and educate patients on alarm symptoms to allow for prompt recognition of life and limb-saving treatments. Orthopedic surgeons should always have a high index of suspicion for ACS and should be aware of the relationship between rhabdomyolysis and ACS.

## Introduction

Surgical site infections (SSIs) are a common complication seen after inpatient procedures. They remain a major cause of patient morbidity and create a significant financial burden on the healthcare system. It is estimated that two to four percent of patients undergoing inpatient procedures are affected by SSIs, with three percent of this group ultimately succumbing to their infected wounds [1]. SSI risk increases with patient-related factors, including diabetes, older age, obesity, tobacco use, malnutrition, and colonization with microorganisms like Staphylococcus aureus [2]. Over 25% of hospital-acquired infections in the U.S. can be attributed to an implantable device [3]. In particular, surgical implants are difficult to treat as they typically require prolonged treatment with extra surgical procedures and an extended course of antibiotics [4]. The increasing geriatric population, the technological advances utilizing implants, and the ongoing battle against drug-resistant bacteria, SSI prevention, and prompt recognition are vital.

Daptomycin is an antibiotic commonly utilized against gram-positive organisms. The Federal Drug Administration approves it for complicated skin and soft tissue infections, bacteremia, intravascular infections, and osteomyelitis. Daptomycin binds to the cell membrane of susceptible organisms and causes rapid depolarization of membrane potential. A rare but life-threatening complication of daptomycin is rhabdomyolysis, a disorder of rapid skeletal muscle breakdown leading to the release of intracellular components. One study found that daptomycin was responsible for the most drug-induced rhabdomyolysis compared to other antibiotics known to cause this complication [5]. Rhabdomyolysis can cause significant patient morbidity, including renal failure, disseminated intravascular coagulation, and acute compartment syndrome (ACS). Prompt recognition and early treatment of acute compartment syndrome are vital to prevent limb and life-threatening complications [6]. We present a case of daptomycin-induced rhabdomyolysis leading to bilateral forearm acute compartment syndrome in a patient treated for an infected penile implant.

## Case presentation

The patient is a 55-year-old male with a past medical history of prostate cancer, chronic pulmonary embolisms, recurrent DVTs, morbid obesity, type two diabetes mellitus, sarcoidosis, and polysubstance abuse who presented to the emergency department with complaints of acute onset bilateral upper extremity swelling. The patient was six weeks out from a penile implant prosthetic surgical procedure performed at an outside hospital. At four weeks postoperatively, he developed methicillin-sensitive Staphylococcus aureus (MSSA) bacteremia and a methicillin-resistant Staphylococcus aureus (MRSA) infection of his penile implant. Incision and drainage was performed on the patient's scrotum at another hospital. After his debridement, wound cultures from the penile implant no longer demonstrated MRSA. However, he continued to test positive for MSSA bacteremia. He was sent home with four weeks of daily IV-daptomycin infusion via a peripherally inserted central catheter (PICC).

**Figure 1:**
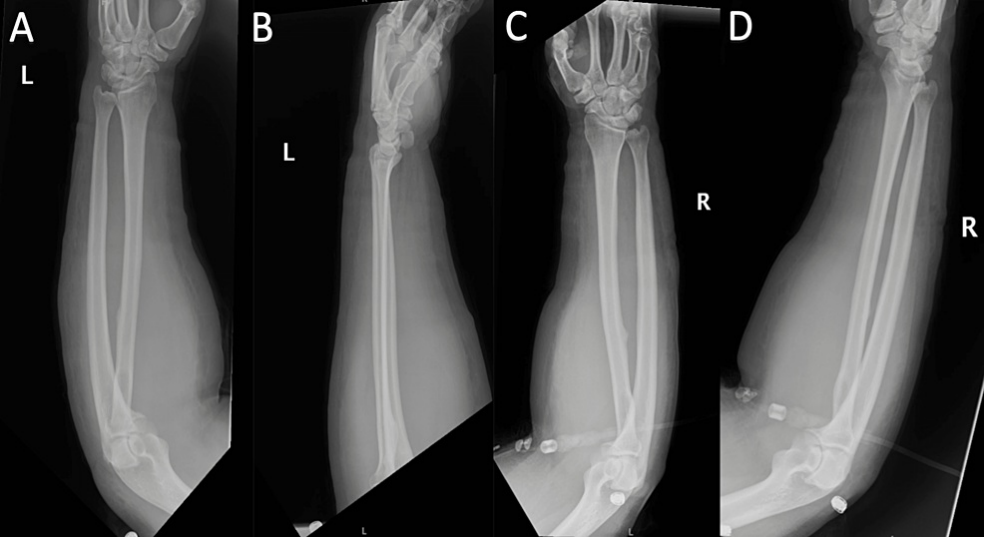
(A) Left forearm AP view, (B) Left forearm lateral view, (C) Right forearm AP view, and (D) Right forearm lateral view, all showing diffuse soft tissue swelling with intact osseous structures. AP: anteroposterior

The patient presented to our emergency department five days after daptomycin initiation, complaining of substernal chest pain, bilateral forearm swelling, and discomfort left worse than right. The patient first noticed mild swelling of the left forearm two days after daptomycin initiation that progressively worsened and developed in both arms. On admission, his creatinine kinase (CK) levels were 33,550 IU/L (reference range: 30-220 IU/L), and myoglobin levels were 9965 ng/mL (reference range: 0-90 ng/mL). Creatinine and electrolytes were all within normal limits. Urinalysis revealed brown-colored urine that was positive for 3+ proteins, and urine toxicology was positive for tetrahydrocannabinol (THC) and tricyclics. XR imaging (Figures 1A-1D) of bilateral forearms showed intact bony structures without signs of acute fracture but with diffuse soft tissue swelling.

The patient was admitted and started on IV hydration for daptomycin-induced rhabdomyolysis, and daptomycin infusion therapy was discontinued. Orthopedic surgery was consulted, and Stryker Intracompartmental Pressure Monitor System needle measurements were obtained. These yielded left forearm pressures of 45 mmHg in the volar compartment and 16 mmHg in the dorsal compartment (reference range: 0-8 mmHg, critical pressure > 30 mmHg) to support the diagnosis of compartment syndrome. All test results obtained with reference values are summarized in Table 1.

**Table 1:** Patient's laboratory values and test results.

Test Result	Patient Value	Reference Range
Creatinine Kinase (CK)	33,550 IU/L	30-220 IU/L
Myoglobin	9,965 ng/mL	0-90 ng/mL
Forearm Volar Compartment Pressure (Left)	45 mmHg	0-8 mmHg, critical pressure > 30mmHg
Forearm Dorsal Compartment Pressure (Left)	16 mmHg	0-8 mmHg, critical pressure > 30mmHg

Within an hour of evaluation by orthopedic surgery, his forearm pain acutely worsened. Despite positive measurements only in his left forearm, his right forearm was also clinically tense. He was taken to the OR for uncomplicated emergent fasciotomies of bilateral volar forearms. The underlying muscle revealed contractures but was intact without any signs of necrosis. Fasciotomy incision sites were left open and monitored with wet-to-dry dressing changes. A second look procedure was performed three days later, where fasciotomy wounds were irrigated, debrided, and surgically closed without complication. The patient was discharged on IV-cefazolin infusion via his PICC line every eight hours for his persistent MSSA bacteremia. Follow-up with orthopedic surgery was done two weeks postoperatively from the initial fasciotomy, demonstrating well-healing bilateral forearm surgical wounds and mild transient ulnar paresthesia in the right upper extremity. Six days after the completion of IV-cefazolin infusion, his bacteremia showed complete resolution on repeat blood cultures. Formal occupational therapy was ordered at this visit with the plan to follow up in four weeks; however, follow-up was lost with the patient.

## Discussion

Daptomycin, a cyclic lipopeptide derived from Streptomyces roseosprous, is an antibiotic commonly deployed against complicated gram-positive bacterial infections [7]. Compared to other available antibiotics, daptomycin carries one of the highest rates of rhabdomyolysis as a possible drug-induced complication [5]. Rhabdomyolysis is the breakdown of skeletal muscle integrity that can range from asymptomatic to the triad of myalgias, limb weakness, and myoglobinuria [8]. However, this triad is seen in less than 10% of patients [9]. This leaves blood CK levels as the most sensitive marker for rhabdomyolysis monitoring, with CK levels ranging anywhere from 10,000 to 200,000 U/L [10]. The breakdown of muscle cells releases potentially toxic substances such as electrolytes, myoglobin, and sarcoplasmic proteins into circulation. Possible electrolyte derangements include hyperkalemia, hyperphosphatemia, hyperuricemia, and even hypocalcemia in extreme cases [8]. Other known causes of rhabdomyolysis include trauma, muscle compression, alcohol abuse, and drugs like cocaine. Often there is not a single cause of rhabdomyolysis, but rather it is the multiplicative effect of many contributing factors that lead to its development [10]. Rhabdomyolysis can progress to both early and late complications. Some early complications include electrolyte abnormalities, arrhythmias, cardiac arrest, and hepatic inflammation. Late complications are acute renal failure caused by myoglobin and disseminated intravascular coagulation from releasing thromboplastin and prothrombotic substances. One severe time-independent complication of rhabdomyolysis is acute compartment syndrome [6].

Acute compartment syndrome is a disruption of perfusion within an osteofascial compartment from increased intracompartmental pressure [11]. The 5 P's (pain, pallor, pulselessness, paralysis, paresthesias) are taught as the presentation of ACS. However, pulselessness and paralysis are rare sequelae that present in later stages [12]. Patients commonly complain of "pain out of proportion" with passive muscle stretching. The reduced blood flow begins to occur at pressures above 30 mmHg, which can be detected by a Stryker compartment pressure monitor. Common causes of ACS include fractures, crush injuries, burns, and infections, with ACS developing most commonly in the anterior compartment of the lower extremity after tibial shaft fractures [11]. Limb-saving treatment requires emergent fasciotomy. In severe cases of rhabdomyolysis, the large release of electrolytes, myoglobin, and creatinine kinase can overwhelm surrounding muscle tissues, increasing the intracompartmental pressure and compromising the vasculature [13]. Untreated acute compartment syndrome can progress to permanent muscle damage, severe kidney damage, electrolyte abnormalities, infection, amputation, or death [11-12]. Conversely, severe ACS can lead to the progression of rhabdomyolysis. Both syndromes together can further perpetuate one another with increasing CK levels. 

Our patient endorsed bilateral forearm swelling and discomfort two days after initiating IV-daptomycin. On day five of daptomycin therapy, the patient presented to our ED due to worsening bilateral forearm swelling and chest pain. Upon admission, his CK levels were 33,550 U/L (reference range: 30-220 IU/L), myoglobin levels were 9965 ng/mL (reference range: 90 ng/mL), his urine was brown, and the patient was experiencing muscle weakness and pain in his bilateral arms. This was a clear diagnosis of rhabdomyolysis and bilateral forearm ACS. It is difficult to determine the exact timeline of his disease progression given that the patient's CK and myoglobin levels were not measured until five days after he began daptomycin therapy, and the bilateral forearm swelling had begun three days prior. Whether he developed daptomycin-induced rhabdomyolysis that progressed to bilateral forearm ACS or initially developed ACS that progressed to rhabdomyolysis could be of question. ACS-induced rhabdomyolysis is unlikely, given that the patient had no history of bilateral forearm trauma or localized forearm infection that could cause ACS. Although daptomycin-induced rhabdomyolysis is rare, a well-documented and known adverse effect makes this the most likely etiology for the progression to ACS.

Additionally, the patient had a history of polysubstance abuse, including cocaine use. A urine toxicology screening weeks before the patient's penile prosthetic surgery was positive for THC and cocaine. Urine toxicology on ED admission four weeks postoperatively was positive for THC and tricyclics; however, it is unclear when the patient discontinued cocaine use between this timeframe. According to the American Addictions Center, cocaine can be detected in the urine anywhere from three days to two weeks [14]. As rhabdomyolysis is often the result of multiple factors, one could speculate that any recent cocaine use on top of the daptomycin infusion could have further contributed to the development of this complication [15].

## Conclusions

Daptomycin infusion is a rare but serious cause of drug-induced rhabdomyolysis. Both severe rhabdomyolysis and acute compartment syndrome have the propensity to lead to one another. Proper management within a multidisciplinary team is vital to prevent severe complications from these events. Orthopedic surgeons should be aware of the relationship between rhabdomyolysis and acute compartment syndrome and have a high index of suspicion for any patient presenting with pain out of proportion in a limb.
